# Characterisation of the *Arabidopsis thaliana* telomerase TERT-TR complex

**DOI:** 10.1007/s11103-024-01461-w

**Published:** 2024-05-14

**Authors:** Barbora Štefanovie, Leon P. Jenner, Lucie Bozděchová, Petr Fajkus, Eva Sýkorová, Jiří Fajkus, Jan J. Paleček

**Affiliations:** 1 Faculty of Science, National Centre for Biomolecular Research, Masaryk University, Kamenice 5, 62500 Brno, Czech Republic; 2 Mendel Centre for Plant Genomics and Proteomics, Central European Institute of Technology, Masaryk University, Kamenice 5, 62500 Brno, Czech Republic; 3 Institute of Biophysics, The Czech Academy of Sciences, Kralovopolska 135, 61200 Brno, Czech Republic

**Keywords:** *A.thaliana* telomerase, AtTERT, AtTR, Protein-RNA interactions, Yeast three-hybrid

## Abstract

Most eukaryotic organisms employ a telomerase complex for the maintenance of chromosome ends. The core of this complex is composed of telomerase reverse transcriptase (TERT) and telomerase RNA (TR) subunits. The TERT reverse transcriptase (RT) domain synthesises telomeric DNA using the TR template sequence. The other TERT domains contribute to this process in different ways. In particular, the TERT RNA-binding domain (TRBD) interacts with specific TR motif(s). Using a yeast 3-hybrid system, we show the critical role of *Arabidopsis thaliana* (At) TRBD and embryophyta-conserved KRxR motif in the unstructured linker preceding the TRBD domain for binding to the recently identified AtTR subunit. We also show the essential role of the predicted P4 stem and pseudoknot AtTR structures and provide evidence for the binding of AtTRBD to pseudoknot and KRxR motif stabilising interaction with the P4 stem structure. Our results thus provide the first insight into the core part of the plant telomerase complex.

**Supplementary Information:**

The online version contains supplementary material available at 10.1007/s11103-024-01461-w.

## Key message

Universal TRBD and embryophytes-specific KRxR motifs of *Arabidopsis thaliana* telomerase reverse transcriptase (AtTERT) bind the P4 stem and pseudoknot structures of the recently identified RNA subunit.

**Supplementary Information:**

The online version contains supplementary material available at 10.1007/s11103-024-01461-w.

## Introduction

Chromosomes transmit cellular genetic information to subsequent cell generations. One particular area of eukaryotic chromosomes that has received a lot of attention is the telomeres, repetitive non-coding DNA sequences at chromosome ends that form protective structures, including G-quadruplexes (Paeschke et al. 2005) and t-loops (Griffith et al. 1999), and complexes with protective proteins, exemplified by mammalian shelterin (Lange 2005). Telomeres prevent the loss of genetic information during the incomplete replication of chromosome ends and avoid chromosome ends being misidentified as damaged DNA, although this latter function may itself allow genuine DNA damage in telomeres to accumulate (Procházková Schrumpfová et al. 2019). Terminal parts of telomeres that suffer from incomplete replication by conventional replication machinery can be re-synthesised and maintained by the ribonucleoprotein telomerase, which in vitro functions with a minimum of the protein catalytic unit (TERT) and a non-coding RNA template (TR) (Weinrich et al. 1997). However, telomerase assembly and activity in vivo are heavily regulated by organism-specific proteins that bind to telomerase mostly via the TR molecule, and they ensure its activity is exquisitely controlled developmentally and tissue-specifically (Schrumpfová and Fajkus 2020). While the TERT subunit is highly evolutionarily conserved, the TR subunit, providing both the template for the telomeric repeat synthesis and scaffold for telomerase holocomplex assembly, shows diverse sequences, lengths, and biosynthetic pathways in different branches of the tree of life (Fajkus et al. 2021, 2023; Peska et al. 2021).

The most conserved and functionally important TERT domains are the reverse transcriptase (RT) and telomerase RNA-binding domains [TRBD; (Rouda and Skordalakes 2007)]. The RT domain transcribes the TR template sequence into DNA, elongating chromosome ends. The TRBD binds the branch structure ahead of the template sequence and other TR elements, depending on the organism (Jansson et al. 2015). For example, human hTRBD binds to branch and pseudoknot (PK) elements (Wan et al. 2021; Liu et al. 2022). In addition, the human CR4/5 stem element stimulates telomerase activity in trans with PK (Mitchell and Collins 2000; Mason et al. 2003).

Unfortunately, little has been known about telomerase in plants, and even the identity of the model plant *Arabidopsis thaliana* (At) TR was cryptic until recent identification of genuine TRs across land plants and, subsequently, the entire Viridiplantae clades (Fajkus et al. 2019, 2021). Plant TRs share some conserved secondary structure motifs with the other known TRs, e.g., pseudoknot (PK) and stem elements (Song et al. 2019; Fajkus et al. 2021). With this revolution in TR identification, it is now possible to begin identifying the proteins involved in telomerase complex in *Arabidopsis* and other plants. A natural first step is to identify an interaction of TR with the catalytic protein subunit. Although AtTERT was characterised long before AtTR (Fitzgerald et al. 1999; Oguchi et al. 1999), nothing is known so far about the specifics of its binding to AtTR. Therefore, the yeast three-hybrid (Y3H) system was used to map AtTERT and AtTR sites essential for their mutual interactions. Using a range of fragments of the AtTERT protein sequence, AtTR RNA sequence, and their mutations, we report the binding of an AtTERT (aa229-580) TRBD-containing fragment to the predicted PK and stem elements.

## Materials and methods

### Molecular cloning

The full-length (FL) AtTERT and fragments containing aa1-271, aa229-580, aa597-987, and aa958-1123 were published previously (Majerská et al. 2017). AtTERT aa229-580, aa242-580, aa249-580, aa299-580, aa320-580, aa229-575, and aa229-558 were PCR amplified from the aa229-580 construct (primers specified in Table ST1) and ligated into the *Nde*I site of a pGADT7 vector using NEBuilder (New England Biolabs, USA).

The FL AtTR (nts1-262) was PCR amplified from the AtTR/pCRIITOPO plasmid construct (Fajkus et al. 2019) and cloned into either pIIIA/MS2-2 or pIIIA/MS2-1 vectors (Bernstein et al. 2002) using the *SmaI* restriction site to create AtTR-MS2 or MS2-AtTR, respectively (Hybrigenics, USA). Fragments of AtTR nts189-262, nts1-245, and nts25-150 were PCR amplified from the AtTR-MS2 construct (primers specified in Table ST1) and ligated into the *SmaI* site of pIIIA/MS2-2 vector using NEBuilder. The AtTR sequences missing P4 stem (nts179-187 and nts241-249) were PCR amplified from synthetic DNA (Eurofins Genomics, Germany).

The QuikChange Lightning Site-Directed Mutagenesis Kit (Agilent Technologies, USA) was used to create mutations and/or deletions in the pGADT7-AtTERT (aa229-580) or AtTR-MS2 constructs. The sequences of primers used for mutagenesis are listed in Table ST1. PK (P2 + P3) mutations in the FL AtTR-MS2 construct were introduced in two steps. Firstly, the P2 mutation was generated using primer set prBS019 + prBS020. The P2 AtTR-MS2 construct was then used as a template for the second mutagenesis using primers prBS015 + prBS016. Mutations in P2 represent substitutions of four nts (G86A, G88A, G89U, G90C), and mutations in P3 are substitutions of three nts (C129G, G130A, G133U). The fragment of AtTR nts25-150 carrying P2 + P3 mutations was generated by PCR amplification of the above FL AtTR P2 + P3 mutant and ligated into the *SmaI* site of pIIIA/MS2-2 vector using NEBuilder.

### Yeast three-hybrid system (Y3H)

The Y3H system (SenGupta et al. 1996) illustrated in Fig. S1a was used to map AtTERT-TR interactions. AtTR-MS2 (pIIIA/MS2-2 derived) and Gal4AD-AtTERT (pGADT7 derived) plasmids were transformed into *Saccharomyces cerevisiae* strain YBZ-1 and processed using the same protocol as for the classic Y2H system (Paleček et al. 2019), except for cultivation on selection plates missing leucine, and adenine (–LA; instead of –LT). RNA–protein interaction was monitored by YBZ-1 cell growth on selection plates missing leucine, adenine, and histidine (–LAH). In addition, increasing the concentration of 3-amino triazole (3AT), which competitively inhibits the imidazole glycerol-phosphate dehydratase (product of *HIS3* gene), was used to compare the relative strength of the interactions. Each combination was co-transformed at least three times, and at least three independent drop tests were carried out.

In addition, we used β-galactosidase assay to measure the strength of the interaction (Palecek et al. 2001). Single colonies were inoculated into 100 µL of YPD medium (in triplicates) in a 96-well plate and grown O/N at 28 °C. The next day, OD_600_ was measured by the microplate reader BioTEK Powerwave 340 (Agilent) by diluting 10 µL culture in 90 µL H_2_O. The remaining 90 µL of the cultures were washed in 50 µL of Z-buffer (60 mM Na_2_HPO_4_, 60 mM NaH_2_PO_4_, 10 mM KCl, 1 mM Mg_2_SO_4_, pH 7.0), 25 µL of 0.1% SDS was added, followed by addition of 6 µL of chloroform. Reactions were mixed several times by pipetting up and down and incubated for 15 min at 30 °C. Next, 60 µL of 4 mg/mL ONPG was added to each reaction and mixed. The time of the reaction was recorded. Once the yellow colour appeared, the reactions were stopped by the addition of 120 µL of 1 M Na_2_CO_3_ and mixing. The plate was spined at 2500 × rpm for 2 min to remove cell debris. 100 µL of the supernatants were transferred to a new plate, and OD_420_ and OD_550_ were measured by the microplate reader BioTEK. The Miller units were calculated according to the formula:

$${\text{Miller Units }} = { 1}000 \times ({\text{OD}}_{{{42}0}} - {1}.{75} \times {\text{OD}}_{{{55}0}} ) \, / \, ({\text{T}} \times {\text{V}} \times {\text{OD}}_{{{6}00}} )$$

OD_420_ and OD_550_ are read from the reaction mixture, OD_600_ reflects cell density in the cell suspension, T = time of the reaction in minutes, and V = volume of culture used in the assay in mL.

### *AtTR *in vitro* transcription and purification*

AtTR was synthesised using PCR amplification of the AtTR/pCRIITOPO plasmid to produce a complementary DNA template [primers are listed in Table ST1; (Fajkus et al. 2019)]. This was then transcribed using a T7 RNA polymerase kit (New England Biolabs) according to the manufacturer’s instructions. The RNA transcript was then purified using AMPure magnetic beads (Beckman-Coulter, USA) according to the manufacturer’s protocol, eluted in water, and frozen at – 80 °C until needed. RNA concentrations are estimated based on 260 nm absorbance measured using a NanoDrop ND-1000 spectrophotometer and extinction coefficient values from in silico prediction (ExPaSy ProtParam tool).

### Protein expression and purification

AtTERT fragments encoding sequences identical to those used for Y3H experiments were introduced into a pGEX-4T-3 vector (Cytiva, USA). This vector introduces a glutathione synthase transferase (GST) fusion to the resulting protein. Cloned plasmids were then transformed into *Escherichia coli* BL21 RIL cells (Agilent), and successful transformations were selected for Ampicillin and Chloramphenicol resistance present in pGEX-4 T-3 and pACYC plasmids, respectively. Transformed cultures were grown in LB medium at 37 °C with 180 rpm shaking, infused with 100 µg/mL Ampicillin and 68 µg/mL Chloramphenicol. × After 3 h, growth had reached a point that OD_600_ ≈0.6–0.8, whereupon growth temperature was reduced to 25 °C, and 0.6 mM isopropyl-β-D-1-thiogalactopyranoside was added to induce the cells over the next 16 h. Cell cultures were then harvested by centrifugation and stored at − 80 °C until needed. Frozen pellets were resuspended in 2 mL of 100 mM Tris–HCl, 500 mM NaCl, and 0.2% polyethyleneimine (PEI) pH 8 with wide-spectrum EDTA-free protease inhibitors (Roche, Switzerland). The resuspensions were lysed by sonication and clarified by centrifugation at 20,000 ×g for 10 min. Clarified soluble cell extracts were then bound to GST SpinTrap columns (Cytiva), equilibrated, and washed with the same buffer without PEI or protease inhibitors. Protein was eluted with 100 mM Tris–HCl, 500 mM NaCl with 80 mM glutathione, buffer exchanged using Amicon spin concentrators (Merck-Millipore, USA) to reduce the concentration of glutathione to < 1 mM, and either used immediately or flash-frozen in liquid nitrogen and stored at – 80 °C for short periods of time. Protein concentrations are estimated values based on 280 nm absorbance measured using a NanoDrop ND-1000 spectrophotometer and extinction coefficient values from in silico prediction (ExPaSy ProtParam tool).

### Electrophoretic mobility shift assay (EMSA)

EMSA samples were prepared by incubating AtTR (final concentration 40 nM) with increasing concentrations (0–400 nM) of AtTERT aa229-580 or aa320-580 fragments for 20 min at room temperature in binding buffer (50 mM Tris–HCl, 250 mM NaCl, 10 mM potassium acetate plus 3% glycerol added before loading). 2% agarose gels in Tris acetic acid (TAE) buffer were then run at 90 V for 50 min, stained with Sybr Green II dye for RNA in TAE for 30 min, washed in the same buffer for 10 min, and then fluorescence under blue light excitation was recorded using an Amersham Imager 680 (Cytiva). EMSAs were performed at least three times.

### Reconstitution of telomerase activity

Reconstitution of telomerase activity was performed using TnT® Quick Coupled Transcription/Translation System (Promega, USA). The reaction mixture was made according to the manufacturer’s instructions and mixed with 100 ng of each RNA prepared in vitro (see above) and 100 ng of the TERT plasmid (Fajkus et al. 2019). Samples were incubated at 30 °C for 1 h, and 1 µLl of each reaction was immediately used for the TRAP assay.

### Telomerase repeat amplification protocol (TRAP)

TRAP assay was adapted from (Fajkus et al. 2019) with some modifications. Q5 Hot Start polymerase (New England Biolabs) was used and the reactions were prepared in 25 µL according to the manufacturer’s instructions. The reaction conditions were as follows: incubation at 26 °C for 45 min enables extension of the substrate primer (CAMV) by telomerase followed by the PCR step of the TRAP assay (enzyme inactivation at 95 °C for 5 min and 35 cycles of 10 s at 98 °C, 30 s at 60 °C and 30 s at 72 °C with a final extension at 72 °C for 5 min). Products were analysed by electrophoresis on a 12% polyacrylamide gel and visualised by staining with GelStar Nucleic Acid Gel Stain (LONZA, Switzerland). Primers used for the TRAP assay are listed in Supplementary Table ST1.

### Analysis of protein structures

The AlphaFold AtTERT model AF-Q9SPU7-F1-v1 (Varadi et al. 2022) and hTERT cryoEM structure [PDB: 7TRD; (Liu et al. 2022)] were used. Structural models were analysed and visualised using the PyMOL software (Schrodinger Inc., USA).

## Results

### Analysis of the A. thaliana TERT binding to AtTR

**Fig. 1:**
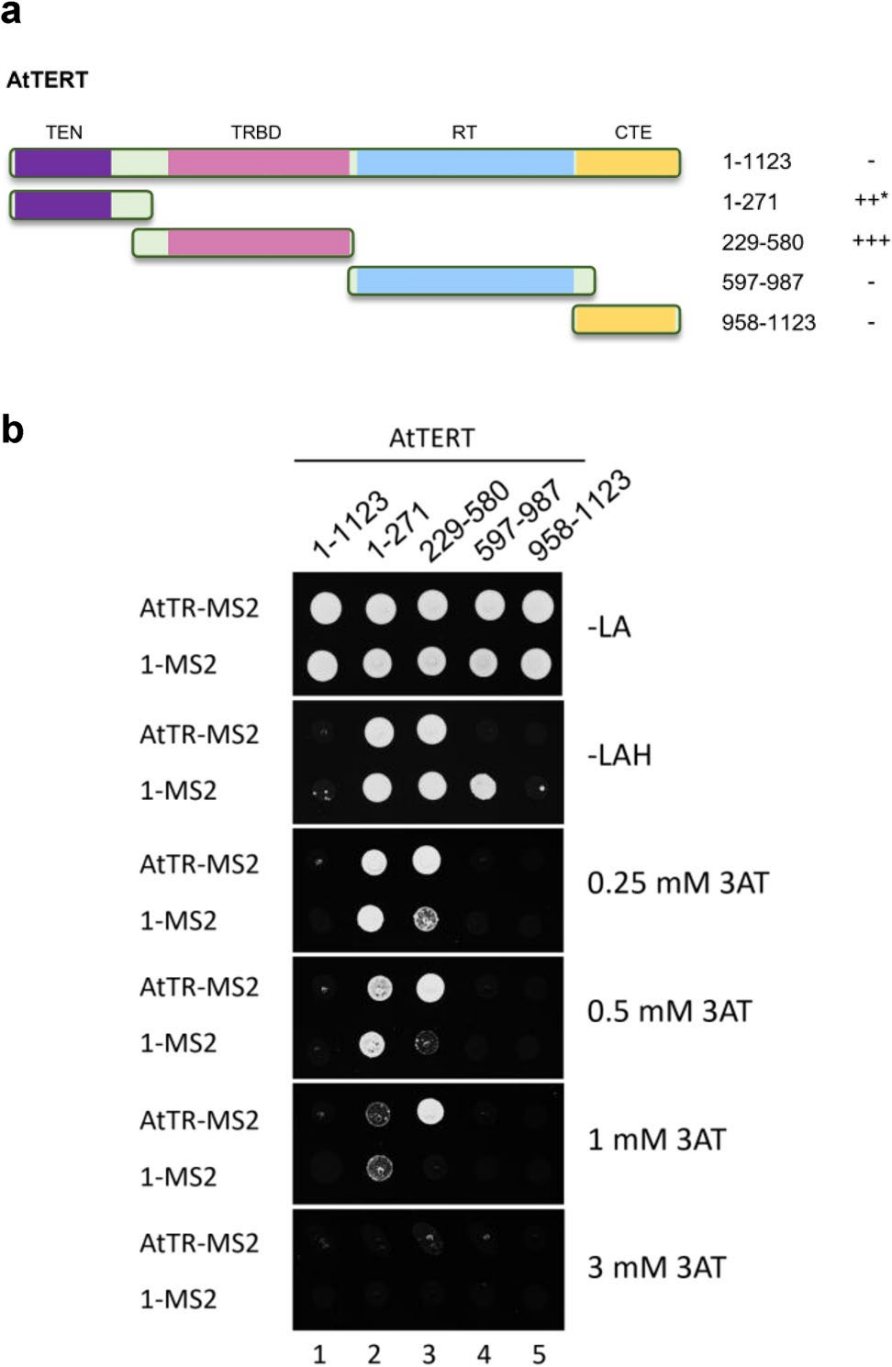
Analysis of the *A. thaliana* TERT binding to AtTR. **a** Schematic overview of AtTERT fragments fused to Gal4AD. Summary of Y3H results from panel b on the right (+ +  + growth on 1 mM 3AT, +  + growth on 0.5 mM 3AT,—no growth on -LAH plates, asterisk marks AtTR-independent binding to RNA). TEN (Telomerase N-terminal, aa11-175; violet), TRBD (Telomerase RNA-Binding Domain, aa299-575; pink), RT (Reverse Transcriptase, aa599-929; light blue), and CTE (C-terminal Extension, aa958-1123; yellow) regions are indicated. **b** Results of the Y3H test, analysing interactions of different AtTERT fragments (panel a) with AtTR-MS2 and 1-MS2 constructs (Fig. S1b). Gal4AD-AtTERT constructs were transformed into *S.cerevisiae* strain YBZ-1 carrying the *HIS*3 reporter gene (Fig. S1a). Interactions were scored by cell growth on -LAH plates with or without the addition of the indicated concentration of 3-amino triazole (3AT). Only the AtTERT fragment containing aa229-580 was able to specifically interact with AtTR-MS2 (lane 3), while the aa1-271 fragment bound RNA unspecifically (with the same affinity to AtTR-MS2 and 1-MS2 control vector; lane 2). A similar binding pattern to AtTERT fragments was detected using the MS2-AtTR construct (Fig. S1c)

To characterise the interactions of AtTERT with newly identified AtTR (Wu et al. 2012; Fajkus et al. 2019; Song et al. 2019), we took advantage of the Y3H system (Fig. S1a), which provides a simple but robust method for studying RNA–protein interactions (Bernstein et al. 2002). Different AtTERT constructs (Zachová et al. 2013; Majerská et al. 2017) were fused with the transcription activation domain of yeast Gal4 transcription factor (Gal4AD-AtTERT), and tested against two hybrid RNA constructs that differed in the order of AtTR and MS2 tandem stem-loops (Fig. S1b). For the initial mapping of the AtTR-interacting site on the AtTERT protein, we used Gal4AD-AtTERT constructs described in (Majerská et al. 2017) harbouring TEN (Telomerase N-terminal), TRBD (Telomerase RNA-Binding Domain), RT (Reverse Transcriptase), and CTE (C-terminal Extension) regions of the AtTERT protein (Fig. 1a). We did not detect any binding of the FL AtTERT to AtTR in either of their combinations (Figs. 1b and S1c, lane 1). Expression testing of Gal4AD-fusion constructs revealed that the FL AtTERT protein was not detectable in the YBZ-1 strain extract (Fig. S1d, lane 1), presumably due to its excessive size. Similarly, we did not detect any AtTR interaction with the aa597-987 fragment harbouring RT domain or CTE fragment (aa958-1123; Figs. 1b and S1c, lanes 4 and 5) despite their detectable signal in the YBZ-1 extracts (Fig. S1d, lanes 4 and 5).

In contrast, the TEN-containing fragment aa1-271 showed interaction with AtTR as well as MS2 controls (Figs. 1b and S1c, lane 2). To exclude the possibility that the Gal4AD-AtTERT(aa1-271) self-activates reporter gene transcription, we transformed all Gal4AD-AtTERT constructs into the L40 parental strain (Palecek et al. 2001), missing the LexADBD-MS2 fusion. As we did not observe any self-activation (Fig. S1e), we concluded that the AtTERT TEN-containing (aa1-271) fragment binds RNA unspecifically in an AtTR-independent manner.

Interestingly, the aa229-580 TRBD-containing AtTERT portion was able to interact with AtTR specifically. This specific interaction was detected for both AtTR-MS2 and MS2-AtTR hybrid constructs on plates containing 1 mM 3AT, while the empty 1-MS2 or 2-MS2 vectors showed only weak background reporter gene activation (Figs. 1b and S1c, lane 3). These data suggest that only the aa229-580 AtTERT fragment containing unstructured (aa176-298) and AtTRBD (aa299-575) regions can specifically bind to AtTR RNA.

### The intact AtTRBD domain is essential for its binding to AtTR

**Fig. 2:**
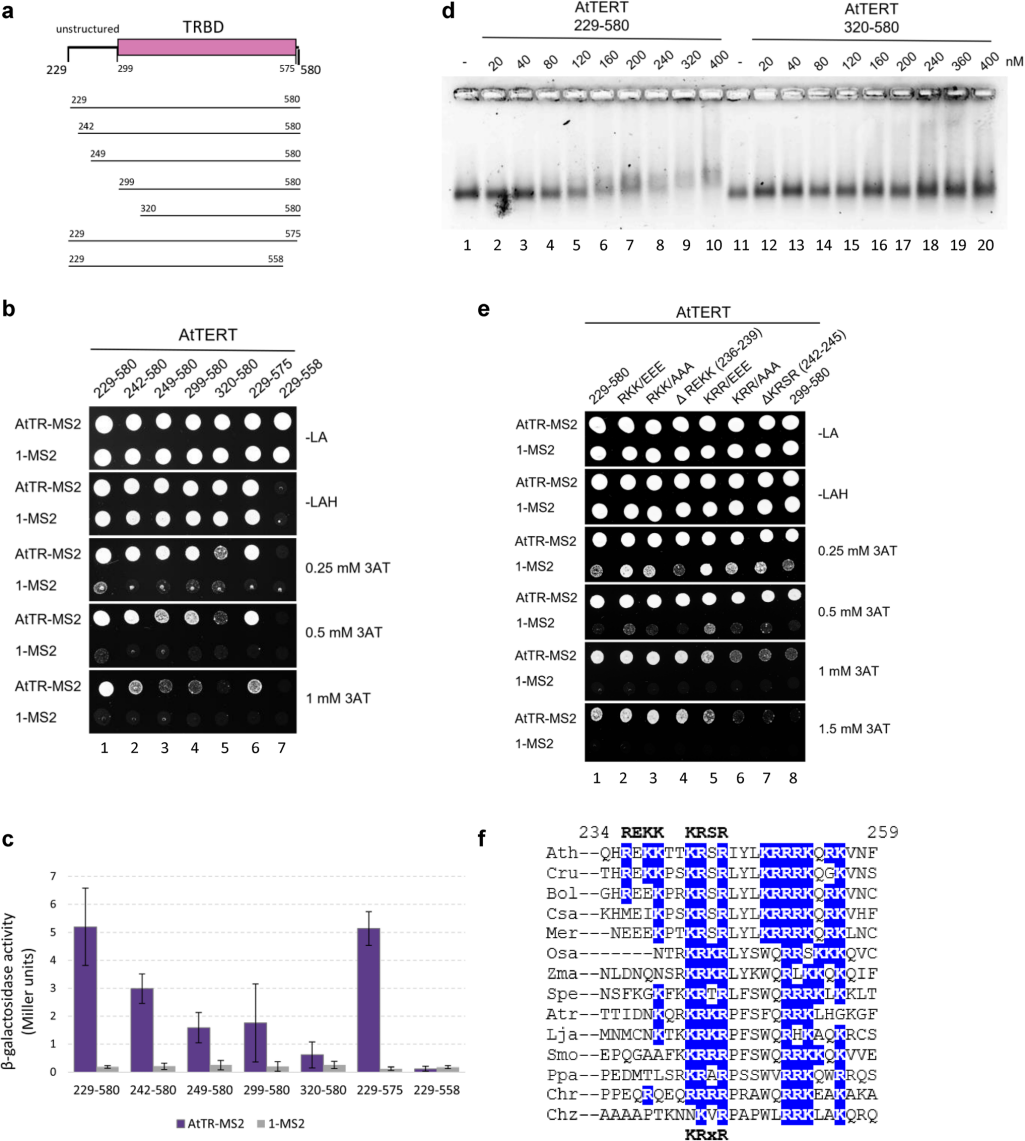
Two AtTERT regions mediate specific interaction with AtTR. **a** Schematic representation of the fragments assayed for AtTR interaction in Y3H. Unstructured (aa229-298) and AtTRBD (aa299-575) regions indicated as in Fig. 1a. **b** *HIS3* reporter gene Y3H results for AtTR-MS2 assayed for interaction with AtTRBD-containing fragments (other Y3H details as in Fig. 1). 1-MS2 vector is used as a negative control. Intact AtTRBD (aa299-575) is critical for the AtTR interaction as its truncations (aa320-580 and aa229-558 fragments) abrogated specific binding to AtTR (lanes 5 and 7). In addition, the aa229-241 and aa242-248 regions contribute to the aa229-580 fragment affinity as aa242-580 and aa249-580 interactions were gradually weaker than the binding of aa229-580 (lanes 1–3). **c** β-galactosidase reporter gene activity results for the interaction of AtTR-MS2 (violet) with AtTERT protein fragments used in panel b. 1-MS2 vector (grey) was used as a control. **d** Representative EMSA results of the purified aa229-580 and aa320-580 AtTERT fragments (Fig. S2c). Increasing concentrations of aa229-580 or aa320-580 proteins were incubated with 40 nM of in vitro synthesised AtTR. Shifts representing the protein-RNA complexes are formed only with the aa229-580 fragment. No binding is apparent for the aa320-580 AtTERT fragment. **e** The Y3H results for the aa229-580 constructs carrying mutations K/R to E or A or deletions (Δ) compared to the aa299-580 fragment (lane 8). The KRSR motif (aa242-245) conserved across embryophyta (panel f) strengthens the stability of the AtTERT-AtTR complex. **f** Sequence alignment of the aa234-259 AtTERT region for the following species: *Arabidopsis thaliana* (*Ath)*, *Capsella rubella (Cru), Brassica oleracea (Bol), Camelina sativa (Csa), Microthlaspi erraticum (Mer), Oryza sativa (Osa), Zea mays* (*Zma), Scilla peruviana (Spe), Amborella trichopoda (Atr), Lygodium japonicum (Lja), Selaginella maellendorffii (Smo), Physcomitrium patens (Ppa), Chlamydomonas reinhardtii (Chr), Chromochloris zofingiensis (Chz). Arabidopsis* REKK and KRSR motifs are labelled above; *blue*, basic residues. The Arabidopsis KRSR sequence is conserved in embryophytes as the KRxR motif (x residue is variable), but it is not present in chlorophytes

To further characterise the AtTR-specific binding of the aa229-580 region, we created several constructs with N- or C-terminal truncations (Figs. 2a and S2a). The C-terminal truncation aa229-575 led to mild weakening of the AtTR interaction (Figs. 2b and c, lane 6). However, further shortening of the AtTRBD to aa229-558 led to the complete loss of AtTR binding (Figs. 2b and c, lane 7), even though the protein level of the aa229-558 fragment was comparable to the protein level of aa229-575 (Fig. S2b, lanes 6 and 7). These data suggest an essential role of the intact AtTRBD (aa299-575) in the AtTERT-AtTR interaction.

Deletion of 13 amino acids from the N-terminus to aa242-580 (Fig. 2a) led to a mild attenuation of the AtTR interaction (Figs. 2b and c, lane 2). Further truncations to aa249-580 or aa299-580 weakened the AtTR interactions to similar levels. Full growth was only observable on 0.25 mM 3AT plates (Fig. 2b, lanes 3 and 4) for these two constructs, and the β-galactosidase levels were the lowest compared to longer (aa229-580 and aa242-580) constructs (Fig. 2c). These data suggest that the aa229-241 and aa242-248 regions play a role in the stability of the AtTERT-AtTR interaction (see below), while the deletion of aa249-298 residues have no effect.

Similarly to the C-terminal truncation of AtTRBD, its N-terminal truncation (fragment aa320-580 of AtTRBD) led to the abrogation of the specific AtTR interaction (Figs. 2b and c, lane 5). To confirm the Y3H results, we used the electrophoretic mobility shift assay (EMSA) approach (Fig. 2d). We purified aa229-580 and aa320-580 AtTERT proteins (Fig. S2c) to test their binding to in vitro transcribed AtTR. The presence of aa229-580 protein retarded the mobility of AtTR with increasing concentration, whereas no mobility change was observed in the presence of aa320-580 protein (Fig. 2d). This result confirms the Y3H data and further corroborates the essential role of the intact AtTRBD (aa299-575) in the AtTERT-AtTR interaction.

To complete the characterisation of the AtTRBD-containing region, we further assessed the contribution of the unstructured (aa176-298) region and protruding helix (aa576-592) linking AtTRBD to RT domain by creating Gal4AD-TERT(aa229-592) and (aa176-592) fragments (Figs. 1a and S2a). Notably, these extensions had no effect on the specific AtTERT-AtTR interaction (Fig. S2d), confirming that the aa229-580 region has the highest affinity to AtTR.

### Embryophyta-specific KRxR motif supports AtTRBD binding to the AtTR

To further characterise the specific contribution of the aa229-241 and aa242-248 unstructured regions to AtTR interaction (Figs. 2b and c), we mutated their basic-rich sequences to acidic glutamate (E) to change the local charge or eliminate the charge by substitutions to alanine. The KRR242, 243, 245/EEE mutations showed slightly impaired interactions with AtTR, and increased non-specific binding to 1-MS2 RNA (Fig. 2e, lane 5). The KRR242, 243, 245/AAA mutant and deletion of the aa242-245 KRSR motif decreased the relative strength of the interaction to a level comparable to the binding of the aa299-580 construct (Fig. 2e, lanes 6–8). This suggests that K242, R243, and R245 residues strengthen the binding of AtTRBD to AtTR (see below).

In contrast, RKK236, 238, 239/EEE, and RKK236, 238, 239/AAA triple mutations did not impair interactions with AtTR compared to wild type (WT) aa229-580, although non-specific binding to 1-MS2 RNA was slightly increased (Fig. 2e, lanes 2 and 3). Moreover, deletion of the aa236-239 region (REKK motif) did not change the strength and specificity of the interaction compared to WT (Fig. 2e, lane 4), even though the protein levels of all triple mutants and deletions were comparable to WT (Fig. S2e). Interestingly, the ^242^KRSR^245^ motif is conserved in embryophytes, while ^236^REKK^239^ is not (Fig. 2f), corroborating further a vital role of K242, R243, and R245 residues for AtTERT-AtTR complex stability.

### The P4 stem AtTR structure binds aa229-298 AtTERT unstructured region and supports telomerase activity

**Fig. 3:**
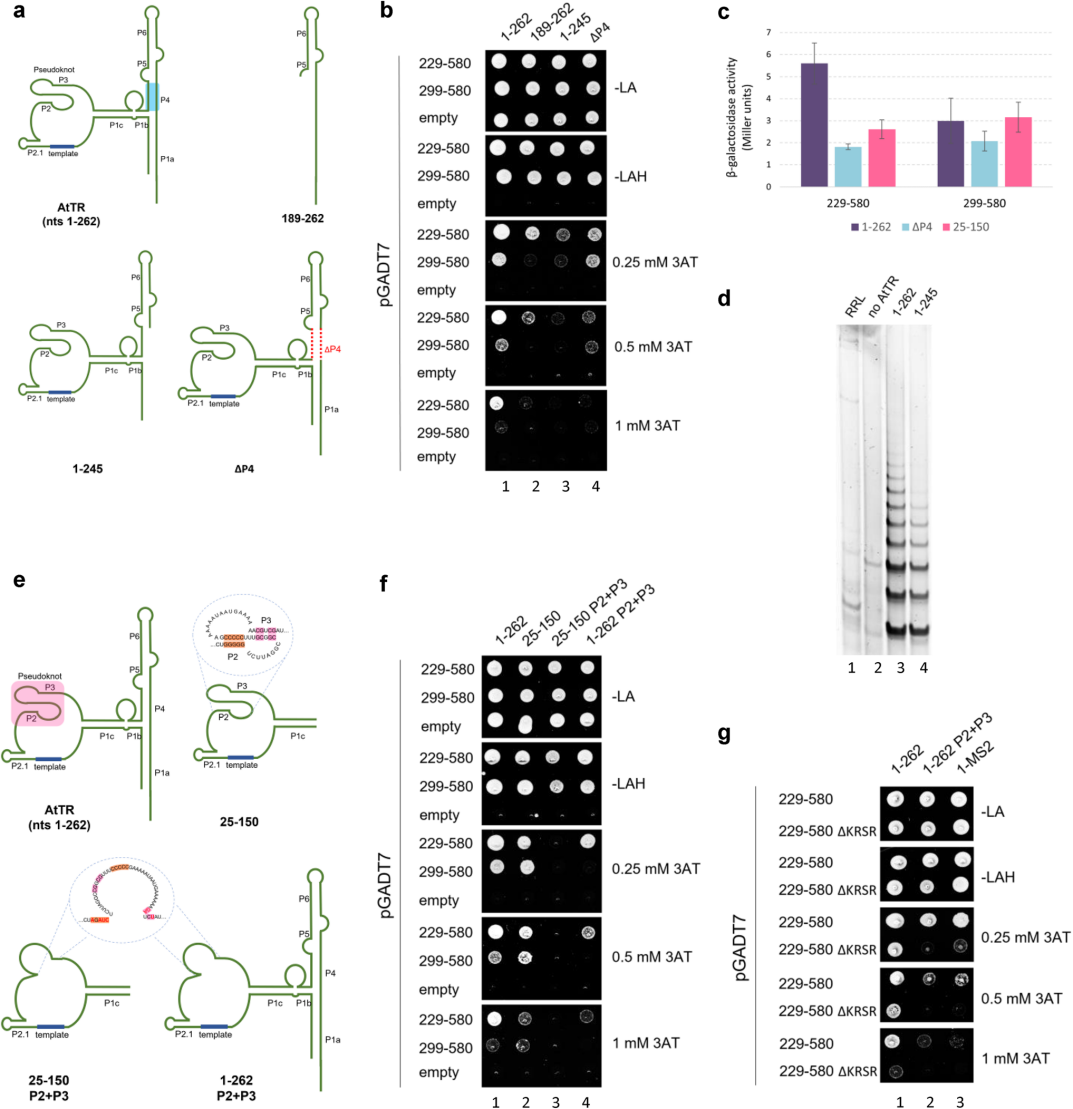
Predicted P4 stem and PK structures are crucial for aa229-580 binding. **a** and **e** Schematic representations of the predicted secondary structure of the FL AtTR molecule and AtTR fragments assayed for their interactions with AtTERT in Y3H (panels b, c, and f). P4 stem element important for binding to AtTERT aa229-580 fragment is highlighted in blue and its deletion (ΔP4) in red. **b** *HIS3* reporter gene Y3H results comparing interactions of the aa229-580 (upper rows) and aa299-580 (lower rows) constructs with the AtTR fragments missing P4 stem structure. **c** β-galactosidase activity results comparing interactions of the aa229-580 (left) and aa299-580 (right) constructs with the FL AtTR (1–262; violet), fragment missing P4 stem (ΔP4; light blue) and PK only fragment (25–150; red). **d** Telomerase Repeat Amplification Protocol results showing FL AtTERT activity with FL AtTR (1–262) and fragment missing P4 stem (1–245). Negative controls (lanes with RRL only and no AtTR) do not contain RNA. **e** Pseudoknot (P2 + P3) is highlighted in pink, and its P2 and P3 sequences are highlighted in orange and pink (top panel). Mutated residues within the pseudoknot region are depicted in red color (bottom panel). **f** Y3H results comparing interactions of the aa229-580 (upper rows) and aa299-580 (lower rows) fragments with the FL (1–262) and PK (25–150) AtTR constructs with or without P2 + P3 mutations of PK. **g** Y3H results comparing interactions of the WT (upper rows) and ΔKRSR (lower rows) sequences of the aa229-580 fragment with the FL AtTR (nts1-262) construct with or without P2 + P3 mutations

To map the AtTR regions important for the AtTERT-AtTR interaction, we created several truncations from the 5′- and/or 3′-end of AtTR (Fig. 3a, unpublished data) and tested them for interactions with aa229-580 and aa299-580 AtTERT fragments using the Y3H system (Fig. 3b). The FL AtTR [nts1-262; (Wu et al. 2012; Fajkus et al. 2019)] showed a relatively strong interaction with aa229-580 (full growth on 1 mM 3AT plate), while binding to aa299-580 was weak (full growth on 0.25 mM 3AT plate; Figs. 3b and c, lane 1). In contrast, the nts189-262 and nts1-245 fragments reduced binding to both AtTERT fragments (Fig. 3b, lanes 2 and 3), suggesting a critical role of the predicted P4 stem structure [Fig. 3a; (Song et al. 2019)]. Therefore, we designed an AtTR construct missing P4 sequences (nts179-187 and nts241-249) within FL AtTR. Deletion of the P4 stem reduced binding to the aa229-580 fragment, confirming the role of the P4 stem in AtTERT-AtTR interaction. Interestingly, binding of ΔP4 to aa299-580 was almost unaffected (Figs. 3b and c), suggesting a role of the unstructured region (presumably KRSR motif; see below) for binding to P4 and interaction of AtTRBD with different AtTR part (presumably PK motif).

To further test the role of the P4 stem structure, we employed the Telomerase Repeat Amplification Protocol (TRAP). Telomerase activity was reconstituted successfully in a rabbit reticulocyte lysate (RRL) combined with in vitro transcribed nts1-262 RNA, producing a ladder of repetitive DNA (Fig. 3d, lane 3). Truncated nts1-245 RNA produced a reduced ladder, suggesting that the AtTERT binding to the P4 stem plays an important role in telomerase activity.

### AtTRBD binds P2-P3 pseudoknot of AtTR

Based on the AtTR secondary structure similarity, previous reports of the human hTERT-hTR complex (Ghanim et al. 2021; Sekne et al. 2022; Bozděchová et al. 2024), and the above results (Fig. 3b), we expected the binding of AtTRBD to the AtTR PK structure. Therefore, we prepared a PK-containing nts25-150 fragment (Fig. 3e) and analysed its binding to aa229-580 and aa299-580 constructs. Consistent with the above results, the binding of aa229-580 to nts25-150 fragment was weaker compared to FL AtTR as the fragment was missing the P4 stem (Fig. 3c and f). Interestingly, binding of the aa299-580 construct to nts25-150 was similar to FL AtTR, consistent with our assumption that AtTRBD binds to the predicted PK motif within FL AtTR.

To test this assumption further, we mutated the PK motif (P2 + P3) within the context of the nts25-150 fragment and FL AtTR (Fig. 3e). Mutations within the PK motif abolished the binding of AtTRBD aa299-580 construct to both nts25-150 and FL AtTR (Fig. 3f, lanes 3 and 4), suggesting that the AtTRBD indeed recognises the PK motif. Interestingly, the aa229-580 construct lost its binding to the mutated nts25-150 fragment with the PK motif no longer available for AtTRBD binding (Fig. 3f, lane 3), while it effectively bound the FL AtTR molecule despite the PK mutation (Fig. 3f, lane 4). These results suggest that the aa229-298 unstructured part (most likely KRSR motif; Fig. 2f) binds to another AtTR motif, most likely P4 (Fig. 3a). Therefore, we tested the deletion of the aa242-245 KRSR motif within aa229-580 fragment against FL AtTR with the mutated PK motif. Combined, the KRSR deletion and PK mutation led to a complete loss of AtTR-AtTERT interaction (Fig. 3g), suggesting that the KRSR motif binds the P4 stem and AtTRBD binds the PK motif.

## Discussion

**Fig. 4:**
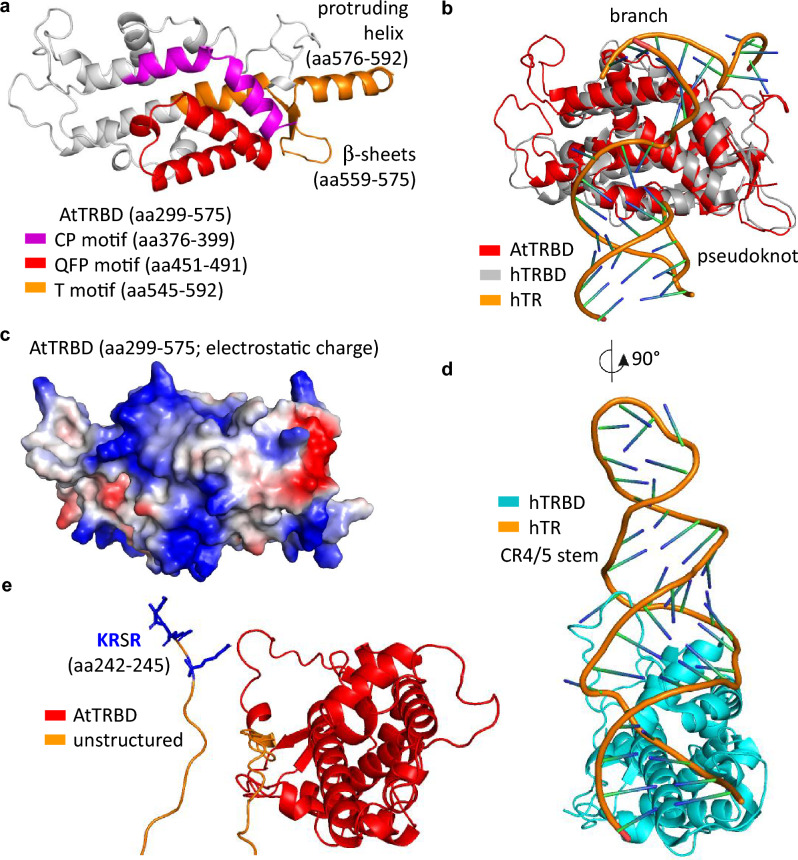
AtTERT model based on the human telomerase structure. **a** An AtTRBD-containing AlphaFold model (aa299-592) with highlighted conserved motifs: CP (pink), QFP (red), and T (orange) (Sýkorová et al. 2006). Two beta-sheets (aa559-575) and protruding helix (aa576-592) of the T motif are labelled (see also secondary structure schema in Fig. S2a). **b** Superposition of AtTRBD (aa299-575; red) AlphaFold model and human hTRBD-hTR cryoEM structure [grey and orange; PDB: 7TRD; (Liu et al. 2022)]. hTRBD binds branch and pseudoknot elements (CR4/5 stem binding is omitted for simplicity). **c** AtTRBD (aa299-575) charge distribution (blue, positive charge) suggests a similar binding pattern of the AtTR molecule (compared to hTRBD-hTR; panel b). **d** Structure of hTRBD bound to hTR CR4/5 stem (PDB: 7TRD; 90° rightward rotated compared to panel b; branch and PK structures are omitted for simplicity). **e.** AlphaFold model of aa242-575 AtTERT part containing unstructured (orange; aa242-298) and AtTRBD (red; 299–575) regions (oriented as hTRBD in panel d). The basic amino acids within the unstructured embryophyta-conserved KRxR motif may stabilise the AtTR P4/P5/P6 stem element binding to AtTRBD (K242, R243, and R245 residues of the KRSR motif are labelled in blue)

Here, we have characterised several motifs critical for the AtTERT-AtTR complex stability. First, we showed the essential function of the intact AtTRBD (aa299-575; Figs. 2b and S2a) for specific binding to AtTR. When we truncated AtTRBD from its C-terminus to residue aa558, binding was lost completely, suggesting the essential role of the highly conserved region, previously annotated as a T motif [aa545-592; (Weinrich et al. 1997; Sýkorová et al. 2006)]. According to structural prediction, this part of the T motif encodes two beta-sheets (aa559-575) and helix (aa576-592; Figs. 4a and S2a). The helix mostly protrudes from the AtTRBD compact structure and links it to the RT domain. Consistent with this arrangement, aa229-580 and aa229-592 construct bound AtTR with the same affinity (Fig. S2d), suggesting that the aa581-592 residues of the protruding helix are dispensable for the stability of the AtTRBD-AtTR complex. In contrast, the two beta-sheets are integral to the AtTRBD structure, and their deletion (in aa229-558 construct) most likely perturbs the structure, abolishing the binding to RNA. Interestingly, the aa229-575 construct bound AtTR with slightly weaker affinity than aa229-580, suggesting a stabilising role of the five aa576-580 amino acids.

The TRBD structure is well conserved across organisms, and its binding to RNA has been described at the molecular level. The ciliate TRBD structure showed binding to the RNA branch site and suggested positioning of the neighbouring template sequence close to the RT pocket [PDB: 5C9H; (Jansson et al. 2015)]. Recent cryoEM structures demonstrated additional binding of PK to TRBD within the human telomerase complex [PDB: 7V99 and 7TRD; (Wan et al. 2021; Liu et al. 2022)], which further stabilises and positions the RNA subunit within the complex (Fig. 4b). Consistent with this, our Arabidopsis AtTRBD-containing constructs bound the nts25-150 fragment containing the predicted branch and PK motifs (Fig. 3). Furthermore, P2 + P3 mutations disturbing predicted PK structure within the nts25-150 fragment completely abolished AtTRBD fragment (aa299-580) binding. These results may explain the previous finding that the region corresponding to the predicted PK structure is essential for telomerase activity (Theimer et al. 2005; Song et al. 2019; Fajkus et al. 2021).

Given the structural and functional conservation of TERT-TR complexes from diverse branches of the tree of life (Wang et al. 2016; Song et al. 2019; Fajkus et al. 2021, 2023), we hypothesise that AtTRBD may bind AtTR in a similar way as human hTRBD-hTR (Fig. 4b). Consistent with this, the most conserved CP and QFP sequence motifs [Fig. 4a; (Sýkorová et al. 2006; Fojtová et al. 2015)] may encode the RNA-binding surface. In particular, the highly conserved QFP motif makes up the basic binding surface for PK (Fig. 4c). Furthermore, the overall basic electrostatic charge distribution at the AtTRBD surface (Fig. 4c) suggests a similar AtTR branch and PK binding path as in the human complex (Fig. 4b).

In addition to PK, TRs contain another conserved structural element comprising a long helical structure with three consecutive short stems [P4, P5, and P6; (Song et al. 2019; Bozděchová et al. 2024)]. This element enables the binding of AtTERT in a manner that is independent of the predicted PK structure, as the aa229-580 construct was able to bind the FL AtTR molecule carrying PK (P2 + P3) mutations (Fig. 3f), while the nts25-150 fragment carrying the same mutations and missing the P4/P5/P6 stem element could not bind. In addition, the constructs missing the P4 sequence disturbed AtTERT-AtTR stability (Fig. 3b), suggesting the important role of the P4 stem-stabilised structure. Accordingly, truncation of the P4 stem part reduced the efficiency of AtTERT telomerase activity (Fig. 3d) and stem structures supported the telomerase activity in trans with PK (Mitchell and Collins 2000; Mason et al. 2003; Song et al. 2019), latter suggesting that the binding site of the stem element is different from the PK binding site.

Consistent with this assumption, the AtTRBD (aa299-580) construct missing the unstructured region (aa229-298) bound FL AtTR with intact PK, but not FL AtTR molecule carrying PK (P2 + P3) mutations (Fig. 3f). This suggests that the binding of the stem element requires this unstructured region. As the location and structure of the plant P4/P5/P6 stem resemble the vertebrate CR4/5 domain (Song et al. 2019), we predict that the P4/P5/P6 stem element binding site at AtTERT is similar to the CR4/5 binding site of the human hTERT (Fig. 4d and e). We assumed that the unstructured KRSR motif may stabilise P4/P5/P6 stem element binding to the AtTRBD site, which is different from the branch-PK binding site (Fig. 4e). Indeed, the combination of the KRSR deletion and PK mutation led to a complete loss of AtTR-AtTERT interaction (Fig. 3g), confirming our assumption that the KRSR motif binds the P4 stem and AtTRBD binds the PK motif. Although the definitive structure of the AtTERT-TR complex remains to be solved by crystallography or cryoEM, our data provide the first and substantial novel insight into the core part of plant telomerase complex defined by interactions between AtTERT and AtTR, and further corroborate their evolutionary conservation.

### Supplementary Information

Below is the link to the electronic supplementary material.

Supplementary file1 (DOCX 18 kb)

Supplementary file2 (PDF 605 kb)

Supplementary file3 (DOCX 32 kb)

## Data Availability

Enquiries about data availability should be directed to the authors.

## Abbreviations

AtTERT: *A.thaliana* TElomerase Reverse Transcriptase
AtTR: *A.thaliana* Telomerase RNA
CTE: C-Terminal Extension
FL: Full-Length
PK: PseudoKnot
RT: Reverse Transcriptase
TEN: TElomerase N-terminal
TRAP: Telomerase Repeat Amplification Protocol
TRBD: Telomerase RNA-Binding Domain
Y3H: Yeast Three-Hybrid
