# Supplementary material for: Characterisation of the Arabidopsis thaliana telomerase TERT-TR complex
Source: Plant Mol Biol. 2024 May 14;114(3):56. doi: 10.1007/s11103-024-01461-w (PMC11093817; doi:10.1007/s11103-024-01461-w)
Supplement: Supplementary file 1 — Supplementary file1 (DOCX 18 kb) [file 11103_2024_1461_MOESM1_ESM.docx]

**Characterisation of the *Arabidopsis thaliana* telomerase TERT-TR complex**

Barbora Štefanovie^1,2^, Leon P. Jenner^3^, Lucie Bozděchová^2^, Petr Fajkus^2,3^, Eva Sýkorová^3^, Jiří Fajkus^1,2,3*^, Jan J. Paleček^1,2*^

^1^ National Centre for Biomolecular Research, Faculty of Science, Masaryk University, Kamenice 5, 62500 Brno, Czech Republic

^2^ Mendel Centre for Plant Genomics and Proteomics, Central European Institute of Technology, Masaryk University, Kamenice 5, 62500 Brno, Czech Republic

^3^ Institute of Biophysics, Czech Acad Sci, Kralovopolska 135, 61200 Brno, Czech Republic

### **Supplementary data:**

### **Supplementary methods**

**Molecular cloning**

AtTERT aa229-592 and aa176-592 fragments were PCR amplified using primers specified in Table ST1. PCR products were ligated into the *Nde*I site of a pGADT7 vector using NEBuilder (New England Biolabs, USA).

**Yeast Three-Hybrid system (Y3H)**

The MS2-AtTR (pIIIA/MS2-1 derived) and Gal4AD-AtTERT (pGADT7 derived) plasmids were transformed into *Saccharomyces cerevisiae* strain YBZ-1 and processed as described in the manuscript.

**Protein analysis**

Protein expression of Gal4AD-AtTERT constructs was assessed as follows. Yeast cells were grown to OD~2.5 and lysed by incubation in 0.1 M NaOH for 5 min and boiling in SDS Laemmli buffer (62.5 mM Tris–HCl, 2% SDS, 5% β-mercaptoethanol, 10% glycerol, 0.002% bromophenol blue; (Kushnirov, 2000)). Samples were separated by 12% SDS-PAGE, blotted, and analysed with anti-HA-HRP (ThermoFisher 26183-HRP or Abcam ab128131) or anti-Gal4AD (G9293, Sigma) antibodies. Anti-HA-HRP antibodies were diluted to 1:5000 in 1% milk-TBST, and incubated for 1 hour at room temperature. Anti-Gal4AD antibodies were diluted to 1:4000 in 1% milk-TBST, incubated O/N at 4 °C, washed in TBST, and incubated with anti-rabbit IgG-Peroxidase secondary antibody (A0545, Sigma) for 1 hour at room temperature. After washing in TBST buffer, chemiluminescence was detected using Super Signal^TM^ West Dura Extended Duration Substrate from ThermoFisher (34075).

The purity of AtTERT fragments expressed in bacteria was analysed on 12.5% SDS-PAGE. Purified protein samples were mixed with SDS Laemmli buffer, separated on PAGE, and stained with Coomassie blue dye.

### **Supplementary Figures**

**Figure S1: a.** Schematic overview of the Y3H system used for the AtTR-AtTERT interaction analysis. Briefly, the first hybrid molecule represents a LexADBD (LexA DNA-binding domain) fused to the bacteriophage MS2 coat protein. The MS2 homodimer protein has a high affinity to a short stem-loop (MS2 RNA) sequence that is a part of the second fusion construct called a hybrid RNA molecule. This hybrid RNA carries two MS2 binding sites and a target RNA molecule, AtTR in this study. The third hybrid construct consists of the transcription activation domain of yeast Gal4 transcription factor (Gal4AD) linked to a candidate RNA binding protein, the telomerase AtTERT subunit in this study. In the case of an interaction between Gal4AD-AtTERT fused protein and AtTR fused to MS2 stem-loop, the hybrid RNA molecule bridges the Gal4AD with the LexADBD, leading to the transcription of the *HIS3* and *LacZ* reporter genes. **b.** Schematic representation of hybrid RNA molecules. Hybrid RNA molecules were constructed by the insertion of the AtTR sequence into either pIIIA/1-MS2 or pIIIA/2-MS2 plasmid vector, providing different orders of AtTR and MS2 tandem stem-loops. **c.** Results of Y3H test using AtTERT fragments in combination with MS2-AtTR construct. Only the AtTRBD-containing fragment (aa229-580) was able to specifically interact with MS2-AtTR (lane 3), similar to AtTR-MS2 (Fig. 1b, lane 3). An empty 2-MS2 vector was used as a control. N-terminal fragment aa1-271 binds RNA in an AtTR-independent way (lane 2). **d.** Expression levels of Gal4AD-fused AtTERT fragments (left). The expected molecular weights are highlighted in bold. Asterisks mark weak bands of aa597-987 and aa958-1123 constructs (lanes 4 and 5). FL AtTERT aa1-1123 expression was not detectable (lane 1). Anti-HA-HRP antibody (26183-HRP, ThermoFisher) in 1:5000 dilution was used for the detection of Gal4AD-fused AtTERT fragments. Loading controls using Ponceau S staining (right). **e.** Self-activation of AtTERT constructs used in Fig. 1b was tested in the L40 parental strain, lacking LexADBD-MS2 fusion. None of the tested constructs showed self-activation.

**Figure S2: a.** Schematic representation of the fragments assayed for AtTR interaction in Y3H (Figs. 2b and S2d). The AlphaFold model (top panel) shows unstructured (aa176-298; orange), AtTRBD (aa299-575; red), and protruding helix (aa576-592; violet) regions. Secondary structures (bottom panel; helix – rectangle, β-list – arrowhead) extracted from the AlphaFold model. **b.** Expression levels of Gal4AD constructs containing AtTRBD domain (left). The expected molecular weights are highlighted in bold. Asterisks mark weak bands of aa320-580, aa229-575, and aa229-558 constructs (lanes 5-7). Anti-Gal4AD antibody (G9293, Sigma) was used in 1:4000 dilution for the detection of Gal4AD-fused AtTERT fragments. Loading controls using Ponceau S staining (right). **c.** Purified GST-AtTERT(aa229-580) and GST-AtTERT(aa320-580) proteins were resolved by 12.5% SDS-PAGE and stained with Coomassie blue dye. **d.** Fragments aa229-592 and aa176-592 (lanes 2 and 3) tested in Y3H for their interaction with AtTR-MS2 showed similar interaction as the aa229-580 AtTERT fragment. **e.** Expression levels of Gal4AD-AtTERT (aa229-580) point and deletion mutants (left). Anti-HA-HRP antibody (ab128131, Abcam) in 1:5000 dilution was used to detect AtTERT mutants.
