# Supplementary figures and images for: Characterisation of the Arabidopsis thaliana telomerase TERT-TR complex
Source: Plant Mol Biol. 2024 May 14;114(3):56. doi: 10.1007/s11103-024-01461-w (PMC11093817; doi:10.1007/s11103-024-01461-w)

Fig. S1

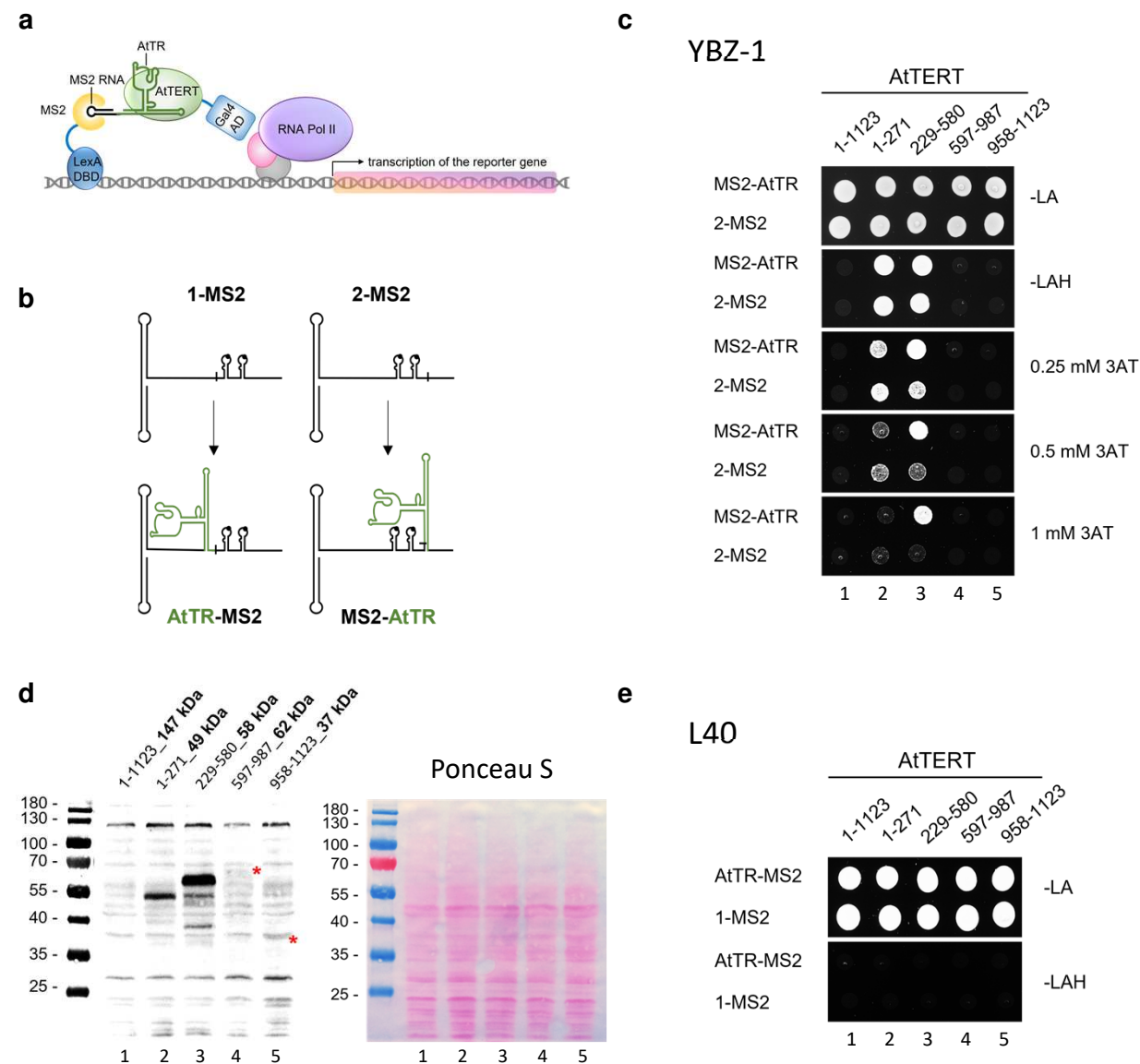

Fig. S2

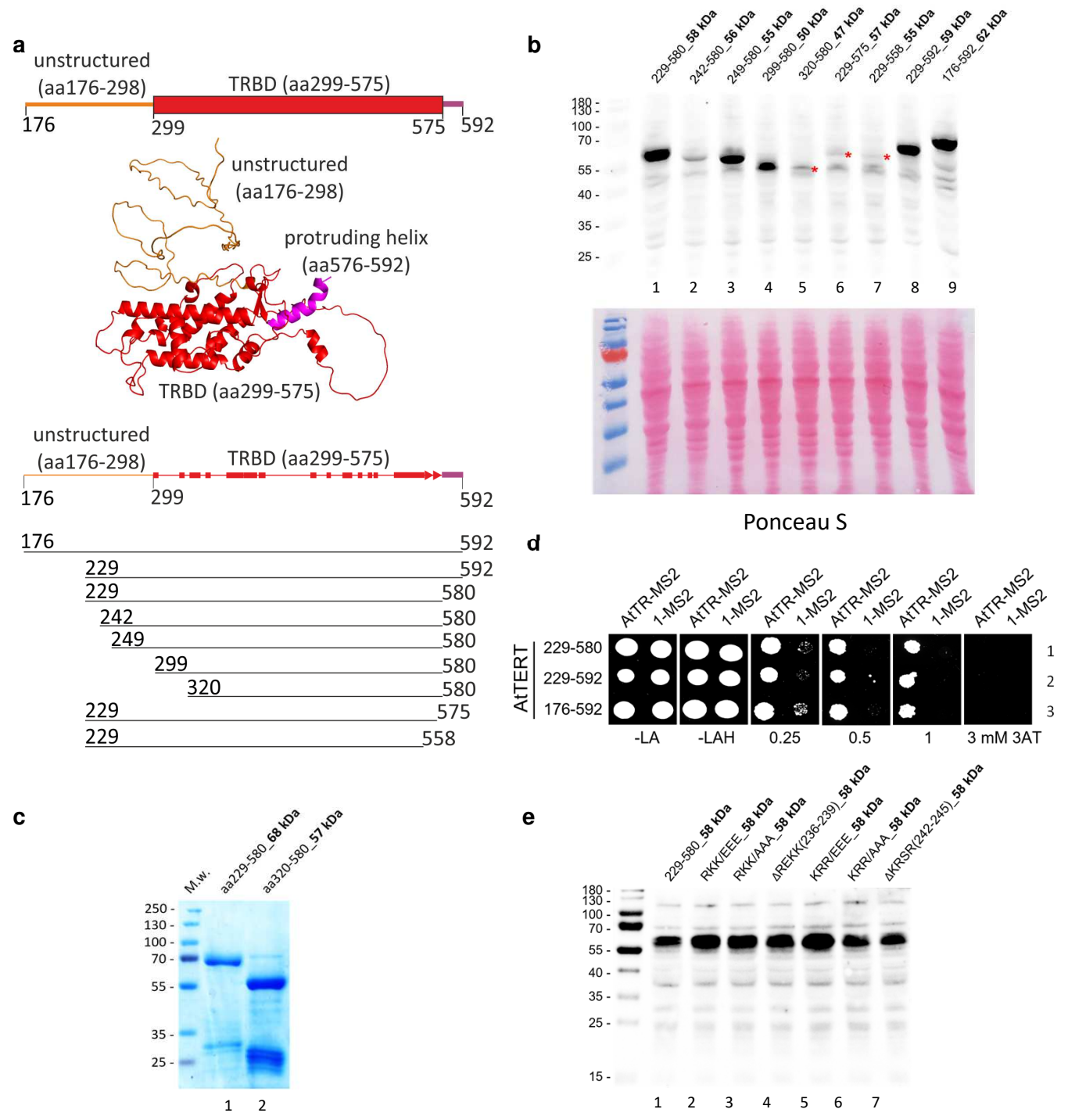

Supplement: Supplementary file 2 — Supplementary file2 (PDF 605 kb) [file 11103_2024_1461_MOESM2_ESM.pdf]
