# Supplementary material for: Characterisation of the Arabidopsis thaliana telomerase TERT-TR complex
Source: Plant Mol Biol. 2024 May 14;114(3):56. doi: 10.1007/s11103-024-01461-w (PMC11093817; doi:10.1007/s11103-024-01461-w)
Supplement: Supplementary file 3 — Supplementary file3 (DOCX 32 kb) [file 11103_2024_1461_MOESM3_ESM.docx]

# Supplementary table

Table ST1 - List of primers used for cloning, mutagenesis, and TRAP assay

| AtTERT aa229-580 to pGADT7 | prBS064 | fw | cccatacgacgtaccagattacgctcatatgggcgaggacgtagacc |
| --- | --- | --- | --- |
|  | prBS065 | rev | gggtggaattcactggcctccatggccaCTAccagctccttttccgg |
| AtTERT aa242-580 to pGADT7 | prBS070 | fw | cccatacgacgtaccagattacgctcatATGAAGCGTTCAAGAATATATC |
|  | prBS065 | rev | gggtggaattcactggcctccatggccaCTAccagctccttttccgg |
| AtTERT aa249-580 to pGADT7 | prBS071 | fw | cccatacgacgtaccagattacgctcatATGAAGCGTCGGCGAAAGCAG |
|  | prBS065 | rev | gggtggaattcactggcctccatggccaCTAccagctccttttccgg |
| AtTERT aa299-580 to pGADT7 | prBS068 | fw | cccatacgacgtaccagattacgctcatATGaagcaggctaaacagg |
|  | prBS065 | rev | gggtggaattcactggcctccatggccaCTAccagctccttttccgg |
| AtTERT aa320-580 to pGADT7 | prBS069 | fw | cccatacgacgtaccagattacgctcatATGgttataccaccaaacc |
|  | prBS065 | rev | gggtggaattcactggcctccatggccaCTAccagctccttttccgg |
| AtTERT aa229-575 to pGADT7 | prBS064 | fw | cccatacgacgtaccagattacgctcatatgggcgaggacgtagacc |
|  | prBS082 | rev | ggaattcactggcctccatggccaCTACCGGTAATAATAAATATTTAGC |
| AtTERT aa229-558 to pGADT7 | prBS064 | fw | cccatacgacgtaccagattacgctcatatgggcgaggacgtagacc |
|  | prBS083 | rev | ggaattcactggcctccatggccaCTAGTTGAAGTGCACCAATTTC |
| AtTERT aa229-592 to pGADT7 | prBS064 | fw | cccatacgacgtaccagattacgctcatatgggcgaggacgtagacc |
|  | prBS079 | rev | gggtggaattcactggcctccatggccaCTAATCAAGGGCTTTGC |
| AtTERT aa176-592 to pGADT7 | prBS081 | fw | cccatacgacgtaccagattacgctcatGGACCTCCTCTATGTATCAAG |
|  | prBS079 | rev | gggtggaattcactggcctccatggccaCTAATCAAGGGCTTTGC |
| AtTERT aa229-580  R236,K238,K239/EEE | prBS125 | fw | ttacgctcatatgggcgaggacgtagaccaacatgaggaggaggagactactaagcgttcaagaatatatcttaagc |
|  | prBS126 | rev | gcttaagatatattcttgaacgcttagtagtctcctcctcctcatgttggtctacgtcctcgcccatatgagcgtaa |
| AtTERT aa229-580  R236,K238,K239/AAA | prBS134 | fw | tatattcttgaacgcttagtagttgcTgcctcTgcatgttggtctacgtcctcgcccatatgagcgta |
|  | prBS135 | rev | tacgctcatatgggcgaggacgtagaccaacatgcagaggcagcaactactaagcgttcaagaatata |
| AtTERT aa229-580  ΔR236,E237,K238,K239 | prBS136 | fw | cttgaacgcttagtagtatgttggtctacgtcctcgc |
|  | prBS137 | rev | gcgaggacgtagaccaacatactactaagcgttcaag |
| AtTERT aa229-580  K242,R243,R245/EEE | prBS097 | fw | cctttctctgctttcgccgacgcttaagatatatctctgactcctcagtagtttttttctctctatgttggtctacatcc |
|  | prBS098 | rev | ggatgtagaccaacatagagagaaaaaaactactgaggagtcagagatatatcttaagcgtcggcgaaagcagagaaagg |
| AtTERT aa229-580  K242,R243,R245/AAA | prBS138 | fw | ctctgctttcgccgacgcttaagatatattgctgaagccgcagtagtttttttctctctatgttggtctac |
|  | prBS139 | rev | gtagaccaacatagagagaaaaaaactactgcggcttcagcaatatatcttaagcgtcggcgaaagcagag |
| AtTERT aa229-580  ΔK242,R243,S244,R245 | prBS140 | fw | ctttcgccgacgcttaagatatatagtagtttttttctctctatgttg |
|  | prBS141 | rev | caacatagagagaaaaaaactactatatatcttaagcgtcggcgaaag |
| AtTR nts189-262 | prBS048 | fw | ccggctagaactagtggatcccccgggCGTAGGTGGTTCTGTTG |
|  | prBS038 | rev | cctgcaggcatgcaagctgcccgggTTTGGGGGTGGGAGGTAAG |
| AtTR nts1-245 | prBS041 | fw | ccggctagaactagtggatcccccgggAGGGGTGTGGGAACCTAG |
|  | prBS056 | rev | cctgcaggcatgcaagctgcccgggAAGGCGAGGAAACGGTTAACC |
| AtTR nts25-150 | prBS037 | fw | ccggctagaactagtggatcccccgggAGTCTGCTTATTGATTGC |
|  | prBS043 | rev | cctgcaggcatgcaagctgcccgggGCCTCTTATGTAGCCATC |
| AtTR nts1-262  P2 | prBS019 | fw | gaaacgccggcctaagagatctagtaattaattcccatagttaacatgagagggt |
|  | prBS020 | rev | accctctcatgttaactatgggaattaattactagatctcttaggccggcgtttc |
| AtTR nts1-262  P2+P3 | prBS015 | fw | cccgaaaaataatgaaaaaagatctatggctacataagaggctcg |
|  | prBS016 | rev | cgagcctcttatgtagccatagatcttttttcattatttttcggg |
| PCR for *in vitro* RNA synthesis nts1-262 | Eva-C98 | fw | TAATACGACTCACTATAGGG AAGGGGTGTGGGAACCTAG |
|  | Eva-C239 | rev | TTTGGGGGTGGGAGGGTAAG |
| PCR for *in vitro* RNA synthesis nts1-245 | Eva-C98 | fw | TAATACGACTCACTATAGGG AAGGGGTGTGGGAACCTAG |
|  | ATR8_245 | rev | AAGGCGAGGAAACGGTTAACC |
| TRAP assay substrate  TRAP assay reverse | CAMV | fw | CGTCTTCAAAGCAAGTGGATT |
|  | TelPr-S | rev | GCCTTAACCCTAAACCCTAAAC |
